# Effects of Olympic Combat Sports on Older Adults’ Health Status: A Systematic Review

**DOI:** 10.3390/ijerph18147381

**Published:** 2021-07-10

**Authors:** Pablo Valdés-Badilla, Tomás Herrera-Valenzuela, Rodrigo Ramirez-Campillo, Esteban Aedo-Muñoz, Eduardo Báez-San Martín, Alex Ojeda-Aravena, Braulio Henrique Magnani Branco

**Affiliations:** 1 Department of Physical Activity Sciences, Faculty of Education Sciences, Universidad Católica del Maule, Talca 3530000, Chile; 2 Carrera de Entrenador Deportivo, Escuela de Educación, Universidad Viña del Mar, Viña del Mar 2520000, Chile; 3 Sciences of Physical Activity, Sports and Health School, Faculty of Medical Sciences, Universidad de Santiago de Chile (USACH), Santiago 9170022, Chile; tomas.herrera@usach.cl (T.H.-V.); esteban.aedo@usach.cl (E.A.-M.); 4 Department of Physical Activity Sciences, Universidad de Los Lagos, Santiago 8320000, Chile; r.ramirez@ulagos.cl; 5 Centro de Investigación en Fisiología del Ejercicio, Facultad de Ciencias, Universidad Mayor, Santiago 7500000, Chile; 6 Department of Sports Sciences, Faculty of Physical Activity and Sports Sciences, Universidad de Playa Ancha, Valparaíso 2340000, Chile; eduardo.baez@upla.cl; 7 Laboratory of Human Performance, Quality of Life and Wellness Research Group, Department of Physical Activity Sciences, Universidad de Los Lagos, Osorno 5290000, Chile; alex.ojeda@ulagos.cl; 8 Faculty of Sports Sciences, Universidad de Castilla-La Mancha (UCLM), 45071 Toledo, Spain; 9 Graduate Program in Health Promotion, Cesumar University (UniCesumar), Maringá 87050-900, PR, Brazil; braulio.branco@unicesumar.edu.br

**Keywords:** martial arts, physical activity, exercise, elderly, aging

## Abstract

The aim of this systematic review was to analyse the studies centered on the effects of Olympic combat sports (OCS [i.e., boxing, fencing, judo, karate, taekwondo, wrestling]) on older adults’ physical-functional, physiological, and psychoemotional health status. The review comprised randomised-controlled trials with OCS interventions, including older adults (≥60 years), and measures of physical-functional, physiological, and/or psychoemotional health. The studies were searched through SCOPUS, PubMed/MEDLINE, Web of Science, PsycINFO, and EBSCO databases until 5 January 2021. The PRISMA-P and TESTEX scales were used to assess the quality of the selected studies. The protocol was registered in PROSPERO (code: CRD42020204034). Twelve OCS intervention studies were found (scored ≥ 60% for methodological quality), comprising 392 females and 343 males (mean age: 69.6 years), participating in boxing, judo, karate, and taekwondo. The qualitative analysis revealed that compared to controls, OCS training improved muscle strength, cardiorespiratory capacity, agility, balance, movement, attention, memory, mental health, anxiety, and stress tolerance. Meta-analysis was available only for the chair stand test, and an improvement was noted after OCS training compared to control. In conclusion, OCS interventions improves older adults’ physical-functional, physiological, and psychoemotional health. Our systematic review confirms that OCS training has high adherence (greater than 80%) in older adults.

## 1. Introduction

Aging is associated with several physical, physiological, and functional changes that are linked to an increased risk of chronic diseases, functional decline, and premature mortality [1]. This process causes loss of muscle mass and muscle strength, increased subcutaneous fat, decreased joint mobility and flexibility, decreased ventilatory capacity, loss of balance and agility, bone decalcification, increased blood pressure, and decreased cognitive functions [2,3,4,5]. These declines, as a whole, have a negative impact on the general well-being and quality of life perception of older adults [6].

Physical activity (PA) is regarded as an essential element for achieving a healthy aging process [6,7,8] and is distinguished as the cheapest alternative of preventive healthcare [9]. These facts, among other reasons, have allowed public and private institutions to implement PA programs aimed at older adults around the world [4,5,6]. International recommendations concerning PA for older adults agree on incorporating exercises that favor different physical capacities, especially endurance and muscle strength, cardiorespiratory capacity, flexibility, agility, and balance [3,8,10,11,12,13]. These exercises must be adapted to the older adults’ characteristics, and their intensity, volume, and duration must be individually defined to achieve maximum benefits [3,10,13]. Diverse studies regarding older adults report significant improvements at physical-functional, physiological, and psychoemotional levels through PA interventions based on endurance and muscle strength training [3,14] and multi-component training [12,15]. Nonetheless, interventions based on sports—with and without rules modification—have reported results that are not conclusive concerning their benefits at the psychosocial level of older adults [16].

On the other hand, adequately dosed martial arts and combat sports have been described as alternatives to improve one’s health [17]. For example, interventions consisting of six to twelve weeks of tai chi have induced significant improvements for cognitive, functional, and metabolic function, and reduce pain perception and improve mental health and sleep quality in men and women of different ages [18,19]. Additionally, a systematic review that includes interventions based on kung-fu, wushu, karate, taekwondo, muay-thai, kickboxing, jujitsu, judo, and kenpo, among others, intended for people over 18 years old, reported positive effects—derived from regular martial arts and combat sports practice—on balance, cognitive function, and mental health. However, the authors propose that the quality of the evidence is affected by methodological deficiencies caused—among other factors—by the fact that half of the studies were cross-sectional [20]. Apparently, martial arts and combat sports are PA strategies that meet the requirements of muscle strength, cardiorespiratory capacity, flexibility, agility, and postural balance that older adults need [18,19,20], as the specific activities of martial arts and combat sports involve attack and defense movements where the upper and lower extremities are used, in addition to choreographies or forms (sequences of arms and legs movements that simulate an imaginary combat) that allow performing dynamic low-impact actions at moderate to vigorous intensities [21].

Therefore, knowing the possible benefits of combat sports with a competitive orientation—such as Olympic modalities—on older adults’ health status from a biomedical perspective through high-quality studies (e.g., randomised-controlled trials) becomes relevant because of their status as dangerous activities [22]. In this sense, the health responses (biopsychoemotional) after Olympic combat sports (OCS) practice were analysed. There has been an increase in productivity in the scientific field that focuses on OCS; for example, since 2004, more than 30 scientific papers regarding judo have been published in the Web of Science each year [23]; this is similar in the case of taekwondo since the year 2011 [24].Having research that synthesises the dosage of exercises and type of activities performed by older adults would support the usage of OCS as safe PA strategies. In this sense, the aim of the present systematic review was to analyse the studies centered on the effects of OCS (boxing, fencing, judo, karate, taekwondo, wrestling) on older adults’ physical-functional, physiological, and psychoemotional health status.

## 2. Methods

The present study followed the preferred reporting guidelines for systematic review protocols and meta-analyses PRISMA-P (Preferred Reporting Items for Systematic Reviews and Meta-analyses Protocols), which correspond to a 17-item checklist intended to facilitate the development and report of a robust protocol for systematic reviews or meta-analyses [25]. The study was registered in PROSPERO (International Prospective Register of Systematic Reviews; code: CRD42020204034).

### 2.1. Eligibility Criteria

The a priori eligibility criteria for this review were the following: (i) original studies written in English, Spanish, or Portuguese; (ii) published from 1 January 1990 to 5 January 2021; (iii) the study included older adults (mean age ≥ 60 years), without sex restriction; (iv) OCS (boxing, fencing, judo, karate, taekwondo, wrestling) interventions with a duration equal to or greater than four weeks; (v) must have a randomised control group with or without supervised PA; (vi) must include at least one physical, functional, physiological, or psychoemotional measured (e.g., muscle strength, postural balance, risk of falling, serum levels, cognitive status, quality of life, etc.) before and after the intervention; and (vii) studies with a randomised-controlled trial design. On the other hand, the exclusion criteria were: (i) cross-sectional, retrospective, and prospective studies, or whose interventions were not centered on OCS; (ii) non-original studies (e.g., letters to the editor, translations, notes, book reviews); (iii) duplicate papers; (iv) review papers (e.g., meta-analyses, systematic reviews, narrative reviews); and (v) case studies (i.e., studies that focus on only one person).

### 2.2. Information and Database Search Process

This review’s research objects are studies centered on OCS interventions effects on older adults’ health status at a physical-functional, physiological, or psychoemotional level. The search strategy process was performed from 24 August 2020, using the SCOPUS, PubMed/MEDLINE, Web of Science, PsycINFO (American Psychological Association) for social and behavioral sciences, and the collection of Psychology and Behavioral Sciences (EBSCO) databases. The medical subject headings (MeSH) from the United States of America National Library of Medicine were used and bias-free language terms related to older adults and OCS. The search string used was the following: (“elderly” OR “older adults” OR “older subject” OR “aging” OR “ageing” OR “aged”) AND (“boxing” OR “fencing” OR “judo” OR “karate” OR “taekwondo” OR “wrestling”). To include the most recent studies in the review, quote alarms were set in the respective databases; thereby, the main researcher automatically received emails regarding the last updates of the search terms used. These updates were received daily (if they were available), and the studies were eligible for their inclusion until the start of the manuscript preparation (5 January 2021). After the formal systematic searches, additional manual searches were performed by consulting grey literature (e.g., conference proceedings), which were taken into account if the complete text was available. In addition, the included studies’ reference lists were reviewed, and prior reviews and meta-analyses were examined to detect studies that were potentially eligible for inclusion.

### 2.3. Studies Selection and Data Collection Process

The studies were exported to the EndNote references manager (version X8.2, Clarivate Analytics, Philadelphia, PA, USA), where they were filtered once again by selecting the title, abstract, and keywords. In some cases, it was necessary to check the full paper. Two authors (P.V.-B., T.H.-V.) independently conducted the selection and data collection processes. The possible discrepancies between the two authors regarding the study conditions were resolved through consensus with a third author (R.R.-C.). Afterward, the full text of the potentially eligible studies was reviewed, and the exclusion reasons of those studies that did not meet the selection criteria were informed. The studies’ data were extracted by two authors independently using a form created through Microsoft Excel (Microsoft Corporation, Redmond, WA, USA).

### 2.4. Methodological Quality Assessment

The objective of this phase was to detect the risk of bias for each of the selected studies. To this end, the Tool for the assEssment of Study qualiTy and reporting in EXercise (TESTEX) scale was used [26]. This instrument is a tool that has been specifically designed for its use in physical exercise-based intervention studies. The TESTEX was used to characterise the studies’ methodological quality and as a possible exclusion criterion [26]. It has a 15-point scale (5 points for study quality and 10 points for reporting) [26]. This process was conducted by two authors (P.V.-B., T.H.-V.)—independently from one another—and a third author (R.R.-C.) acted as referee in the doubtful cases, which then were validated by another author (P.V.-B.).

### 2.5. Data Synthesis

The following data from the selected studies were obtained and analysed: (i) author and publication year; (ii) country of origin; (iii) modality: practiced OCS; (iv) sample: total number of participants, mean age, intervention groups, and gender; (v) activities developed during the intervention; (vi) training volume (total duration, weekly frequency, and time per session); (vii) intensity of the intervention; (viii) analysed variables; (ix) data collection instruments; and (x) main outcomes.

### 2.6. Synthesis Measures for Meta-Analysis

Meta-analyses were performed when three or more studies were available for a given outcome [27]. Pre- and post-intervention means and standard deviations (SDs) for dependent variables were used after being converted to Hedges’s *g* effect size (ES) [28,29]. The inverse-variance random-effects model for meta-analyses was used to allocate a proportionate weight to trials based on the size of their individual standard errors [30] and accounting for heterogeneity across studies [31]. The ESs were presented alongside 95% CIs and interpreted using the following thresholds [32]: <0.2, trivial; 0.2–0.6, small; >0.6–1.2, moderate; >1.2–2.0, large; >2.0–4.0, very large; >4.0, extremely large. Heterogeneity was assessed using the I^2^ statistic, with values of <25%, 25–75%, and >75% considered to represent low, moderate, and high levels of heterogeneity, respectively [33]. Risk of bias across studies was explored using the extended Egger’s test [34], with *p <* 0.05 implying publication bias. To adjust for publication bias, a sensitivity analysis was conducted using the trim and fill method [35], with L0 as the default estimator for the number of missing studies [36]. All analyses were carried out using the Comprehensive Meta-Analysis software (version 2; Biostat, Englewood, NJ, USA). Statistical significance was set at *p* ≤ 0.05.

## 3. Results

### 3.1. Studies Selection

**Figure 1:**
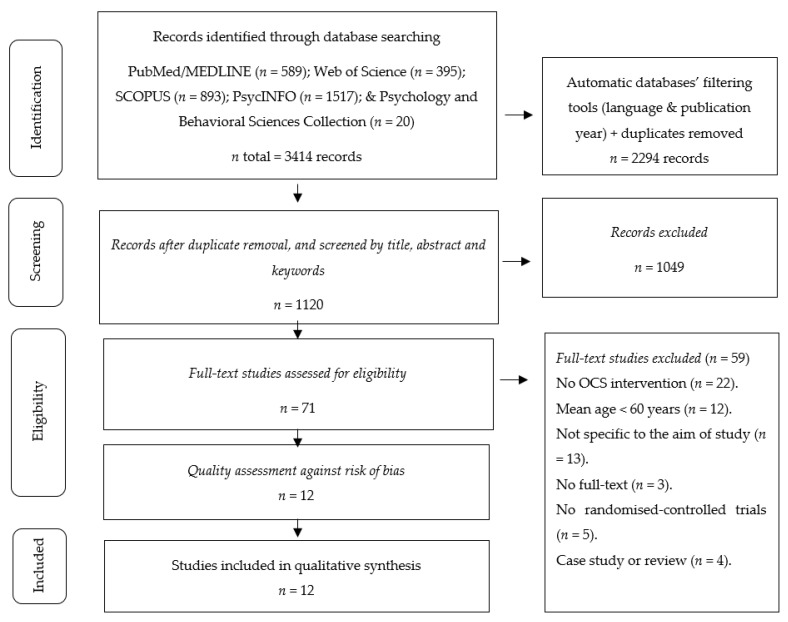
Flowchart of the review process#. #Based on the PRISMA-P recommendations [25].

The search process is detailed in Figure 1. A total of 3414 registers were found in the course of the studies identification stage (PubMed/MEDLINE = 589, Web of Science = 395, SCOPUS = 893, PsycINFO = 1517, EBSCO = 20). During the screening phase, the duplicates were eliminated, and the studies were filtered by selecting the title, abstract, and keywords, thus obtaining 1120 references. The full texts of a total of 71 studies were analysed. Twenty-two studies were excluded because they were not OCS interventions; twelve because the mean age of the sample was less than 60 years of age; thirteen because they did not correspond to the research object, i.e., they were not centered on the health outcomes of older adults; three because the full texts were inaccessible; five because they were not randomised-controlled trials; and four because they were reviews or case studies. After this process, the total number of studies that met the selection criteria was equal to twelve [37,38,39,40,41,42,43,44,45,46,47,48].

### 3.2. Methodological Quality

The selected studies were analysed through the TESTEX scale. Every study obtained 60% or more of the scale’s total score (15 points), as can be observed in Table 1. One study obtained a score of 9/15 [46], two obtained 10/15 [42,45], one obtained 11/15 [37], four obtained 12/15 [41,44,47,48], three obtained 13/15 [38,40,43], and one obtained 14/15 [39].

### 3.3. Studies Characteristics

Five studies were developed in Germany [42,43,46,47,48], two in Italy [38,39], two in South Korea [37,44], one in the United States of America [40], one in China [41], and one in Brazil [45]. As to the practiced OCS modality, two were interventions through boxing [40,41], two through judo [38,39], six through karate [42,43,45,46,47,48], and two through taekwondo [37,44]. No research that used fencing or wrestling as an intervention modality for older adults was found. Table 2 and Table 3 show a summary of the analysed variables for each of the studies selected.

### 3.4. Sample Characteristics

One study had 20 participants [44], five had 30 to 40 [37,38,39,40,45], three had 40 to 70 [42,43,46], and three had more than 80 participants; specifically, Witte, Kropf [48] had 89, Witte, Emmermacher [47] had 90, and Hu, Guo [41] had 198. In total, the sample contained 735 older adults (392 females and 343 males) with a mean age of 69.6 years.

Another characteristic informed by the studies is related to the sample’s initial health level. Seven studies indicate that the participants were functionally independent and without health problems that prevented the practice of PA [40,42,43,45,46,47,48]. Two studies point out that their participants were healthy, functionally independent, with a normal cognitive status [37], and some had mild cognitive impairment [41]. Two studies indicated that their sample was composed of older adults with good physical and mental health and a low risk of falling [38,39]. One study pointed out that its participants were postmenopausal women with stage 2 hypertension [44]. All participants are older adults with no prior OCS experience.

### 3.5. Interventions Conducted and Dosing

As to the intervention groups, six studies had two analysis groups: an experimental group that participated in the OCS intervention and one control group where, in every case, the participants were asked to maintain their usual activities [37,38,39,41,44,45], except for the study by Hu, Guo [41] as the control group activities were not reported. Five studies distributed their sample into three groups: two intervention groups (one through OCS) and one control group [40,43,46,47,48]. As to the second intervention group, four studies described traditional physical fitness protocols that involved exercises and activities in developing endurance fitness and muscle strength, cardiorespiratory capacity, flexibility, agility, and balance [40,46,47,48]. In the study by Jansen, Dahmen-Zimmer [43], the second intervention group was based on mindfulness-based stress reduction, which consists of activities aimed at reducing stress through sitting and walking meditation and exercises of the body, mental states, and emotional awareness. On the other hand, the study by Jansen and Dahmen Zimmer [42] considered four analysis groups (three experimental groups and one control group), specifically: karate group; traditional physical fitness group; cognitive training group intended to improve creative thinking and train fluid intelligence and memory performance through inductive thinking, concentration, and deductive thinking tasks; and a control group, whose activities were not reported.

As to the activities developed through the OCS training protocols, regarding the boxing modality, Combs, Diehl [40] point out that specific cardiorespiratory capacity activities distributed throughout a circuit training were performed, without detailing the exercises, but stating that older adults wore boxing gloves and punching bags without making contact with other people while boxing. Further, Hu, Guo [41], concerning the same modality (boxing), do not specify the activities carried out, only stating that the participants performed shadowboxing (movements through the air without contact). Concerning the judo interventions, these consisted of sessions that started with light routines and dynamic movements with the whole body, imitating judo techniques, followed by specific standing, floorwork, passive, and active techniques which were performed individually and in pairs, ending with specific judo choreographies or forms [38,39]. The karate modality was the most used among the selected studies; in general, the activities consisted of specific movements with the lower (stances and kicking) and upper extremities (punching and blocking) and combinations of both, performed individually and in pairs, in addition to choreographies or specific forms that were adapted to the older adults’ ages [42,43,45,46,47,48]. In addition, Pacheco Lopes Filho, De Oliveira [45] complemented breathing and relaxation exercises through meditation. Moreover, taekwondo interventions were based on basic stances and specific movements with the upper (punching and blocking) and lower extremities (stances, mobilization, and kicking), performed individually and in pairs, in addition to choreographies or specific forms of this modality [37,44]. OCS sessions were led—mostly—by instructors certified and experienced in the modalities described [37,38,39,42,43,44,45,46] or supervised by a professional staff [40,41]. Only two studies [47,48] do not report who led the training.

The duration of the interventions was diverse. One lasted eight weeks [43], six lasted between 12 and 16 weeks [37,38,39,40,44,45], and five lasted between 20 and 24 weeks [41,42,46,47,48]. The training frequency varied between one and five weekly sessions with a duration of 60 [37,38,39,42,43,44,45,46,47,48] to 90 [40,41] minutes per session. Four studies reported the training intensity. Two of them point out that the intensity was moderate to vigorous [38,39], the other two controlled intensity through the participants’ heart rate, specifically, between 30% to 60% [44] and 50% to 80% [37]. Moreover, eight studies do not report the intensity of their interventions [37,41,42,43,45,46,47,48].

### 3.6. Analysed Variables and Data Collection Instruments

The selected studies used different indicators to measure the interventions’ effects. Regarding the physical-functional level, the lower extremities’ strength was assessed through the chair stand test [37,38,47] and one-repetition maximum using a leg extension machine [44]; the upper extremities’ strength was measured through the arm curl test [37,38] and handgrip dynamometer [38,44]. Two studies assessed the flexibility of upper and lower extremities through chair sit-and-reach and back scratch tests [37,38]. Cardiorespiratory capacity was measured through the 2 min step test [37] and 6 min walking test [40]. Agility and dynamic balance were assessed through the timed up-and-go test [37,40], dual-task timed up-and-go test [40], balance ability test [47], and activities-specific balance confidence scale [40]. Stability and gait velocity were assessed through a gait velocity test [40,47] and photocells [39]. In addition, normal walking was measured through the kinematic system for motion analysis [46], hand motor skills through motor task sequence [45], coordination between limbs through a digital metronome [38], a body movement test [41], and motor reaction through the rod test [48]. A study measured daily living activities through a survey [41], and two studies obtained the low risk of falling through the Berg scale [38,40]. Only one study included anthropometric measures such as body mass index, waist circumference, and hip circumference [38].

The physiological level was the least considered among the studies analysed. Two studies used an Enzyme-Linked Immunosorbent Assay kit (ELISA) to measure the serum levels of neurotrophic growth factors [37] and the blood’s catecholamine levels [44]. Additionally, the brachial-ankle pulse wave velocity was obtained using non-invasive arterial tonometry through SphygmoCor [44], and chronic stress was measured through hair cortisol concentration [43].

Measurements concerning psychoemotional levels were diverse. Cognitive functions were obtained through a Mini-Mental State Examination (MMSE) [37,41,45], the dem tect test [48], and stroop colour and word test [37,43]. As to cognitive processing speed, the number–symbol test [42], number-connection test [42,43], and kinematic system for motion analysis [46] were used. Memory performance was measured through the digit-span test [42,43,45], divided attention test [48], mental rotation test [43], and figure test and block-tapping test [42]. Additionally, Pacheco Lopes Filho, De Oliveira [45] considered other assessments to measure memory performance, attention, and cognitive flexibility through the trail making test, Rey-Osterrieth complex figure, Rey auditory-verbal learning test, visual memory span test, Wisconsin card sorting test, verbal fluency test, APT-II attention questionnaire, dysexecutive questionnaire, and prospective and retrospective memory questionnaire. As to psychological aspects, quality of life perception was measured through the Health Survey Short Form (SF-12) version 1 [43] and version 2 [38], and the Parkinson’s disease quality of life scale [40]. A study obtained the body image perception variable through the body image dimensional assessment [38], fear of falling through the falls efficacy scale-international [38], reactive stress tolerance through the determination test version S11 [48], and perceived stress through the trier inventory for chronic stress [43]. Emotional well-being, anxiety, depression, optimism, and pessimism were measured through different assessments; specifically, Jansen and Dahmen-Zimmer [42] used the center of epidemiological studies depressions scale (long version), Pacheco Lopes Filho, De Oliveira [45] utilized the geriatric depression scale and beck anxiety inventory, and Jansen, Dahmen-Zimmer [43] employed the multidimensional mood questionnaire, the hospital anxiety and depression scale, and the life orientation test–revised.

### 3.7. Outcomes

The OCS interventions achieved significant improvements regarding older adults’ physical-functional, physiological, and psychoemotional aspects. However, most studies/outcomes did not qualify for the meta-analysis and were assessed qualitatively.

#### 3.7.1. Physical-Functional Outcomes

There were significant improvements in the tests related to the lower extremities’ muscle strength [37,38,44,47]. Only three studies [37,38,47] provided data for the chair stand test, involving five OCS groups and three control groups (pooled *n* = 157). There was a significant difference in favor of OCS compared to controls on the effect over the chair stand test performance (ES = 0.59; 95% CI = 0.11 to 1.07; *p* = 0.016; I2 = 49.5%; Egger’s test *p* = 0.017). After adjustment for bias according to the sensitivity analysis conducted using the trim and fill method, the values changed to ES = 0.45; 95% CI = −0.07 to 0.97. The relative weight of each study in the analysis ranged from 7.0% to 28.4%. The tests related to the muscle strength of upper extremities, specifically, the arm curl test [37,38] and handgrip test [38,44], presented significant improvements in the OCS groups without providing data for comparison with the control groups.

The tests related to flexibility, for the lower extremities (chair sit-and-reach test) and upper extremities (back scratch test), presented significant improvements in two OCS groups [37,38], without providing data for comparison with the control groups.

Cardiorespiratory capacity [37,40], agility, and balance [40,47] presented significant improvements in the OCS groups without providing data for comparison with the control groups. In addition, significant improvements were presented in the tests related to gait velocity [40], gait stability [39], walking speed [47], step length, and cadence [39,46]. These improvements were achieved by Ciaccioni, Capranica [39] in flat and hurdling conditions. A study reported significant improvements regarding body movement, gait stability, and arm rotation [41]. Motor reaction improved significantly in the study by Witte, Kropf [48], while Ciaccioni, Capranica [38] reported a significant reduction of waist circumference. Further, Hu, Guo [41] reported a significant increase in the daily living activities score.

#### 3.7.2. Physiological Outcomes

No data was available to compare OCS vs. control groups. However, Cho and Roh [37] reported significant improvements regarding the brain-derived neurotrophic factor, vascular endothelial growth factor, and insulin-like growth factor-1 within all the intervention groups. Moreover, Lee, Scott [44] reported a significant decrease in resting epinephrine within the OCS group and a significant increase in norepinephrine within the control group. However, Jansen, Dahmen-Zimmer [43] could not establish a significant correlation between the pre- and post-intervention hair cortisol levels.

#### 3.7.3. Psychoemotional Outcomes

For the tests related to cognitive aspects, no data was available to compare OCS vs. control groups. However, they improved significantly after OCS training in the stroop colour and word test [37,43], attention [37], divided attention [48], dual-task [46], immediate memory and delayed recall [41], visual memory and memory loss reduction [45], cognitive processing speed [43], and executive functions [45].

For the tests related to psychological aspects, no data was available to compare OCS vs. control groups. Two studies [38,43] provided data for the quality of life perception measured through the SF-12 in the physical and mental health aspects, reporting significant improvements in OCS groups. Additionally, there were significant improvements after intervention with OCS for emotional well-being [42], mental health and anxiety [43], and stress tolerance [48]. However, Ciaccioni and Capranica [38] did not observed significant changes in psychological variables after OCS intervention.

### 3.8. Adherence and Drop-Out

Another relevant aspect corresponds to the participants’ adherence and drop-out to the OCS interventions. Two studies do not report drop-out among their participants regarding karate [46] and taekwondo [44] training. Five studies achieved adherence greater than 90% with respect to boxing [41], karate [43,47,48], and taekwondo [37] interventions. Three studies reported participants’ adherence between 80% and 85% for judo [38,39] and karate [42] training. Two studies informed adherence of 64% for boxing [40] and 48% for karate [45]. Further, traditional physical fitness interventions achieved varied adherence results; Combs, Diehl [40] reported 78%, three studies achieved adherence between 80% and 90% [42,47,48], and one study had no drop-out [46]. Moreover, cognitive training achieved adherence of 85% [42], and mindfulness-based stress reduction achieved adherence of 77% [43]. The main reasons for drop-out were related to diseases or personal problems [39,40,45,48], not meeting the training sessions’ minimum attendance [38,45,48], loss of interest [40,41], conflicts with the training schedule [40,48], and foot injuries [41]. Four studies did not report the reasons for their participants’ drop-out [37,42,43,47].

## 4. Discussion

This systematic review aimed to analyse the effects of OCS on older adults’ physical-functional, physiological, and psychoemotional health status. After reviewing 3414 records, twelve studies met the inclusion criteria and scored ≥60% for methodological quality. The main result of our review indicates that regular practice of OCS in older adults produces beneficial changes on physical-functional, physiological, and psychoemotional variables. This strengthens the scientific evidence supporting the use of martial arts and combat sports as PA alternatives to improve the older adults’ health status [18,20,49].

Concerning the physical-functional level, performance results of the upper and lower extremities strength tests only presented significant differences in favor of the OCS groups vs. control groups in the chair stand test [37,38,47], while the arm curl test [37,38] and handgrip test [38,44] improved in the OCS groups without reporting data to compare with the control groups. The benefits observed could be related to the activities promoted by OCS, which mostly involve lower extremities movements (i.e., stances, kicking, and mobilization) and choreographies or specific forms that demand a significant contribution from the thigh and leg muscle groups. The maintenance and improvement of older adults’ muscle strength are related to functional independence [50], which could positively impact their quality of life [14]. The flexibility of upper and lower extremities measured through the chair-sit-and-reach test and back scratch test [37,38] improved in OCS groups, without providing data for comparison with the control groups. This capacity is reduced over the years due to increased stiffness of cartilage and tissues, which reduces the joint range of motion [51]. Cardiorespiratory capacity [37,40], agility and balance [40,47], gait components [39,40,46,47], body movement [41], motor reaction [48], and daily living activities [41] improved, and waist circumference was reduced [38]. However, these variables do not provide data to compare the OCS groups vs. control groups. Improving or maintaining cardiorespiratory capacity, agility, and gait components are essential elements for cardiovascular health and muscle strengthening [6,11], which reduce the impact of sarcopenia. Nonetheless, more evidence is needed to ratify these findings.

The physiological level did not provide data to establish comparisons between the OCS groups and control groups. However, there were benefits at the brain-derived neurotrophic factor [37], and epinephrine [44] levels of the older adults intervened through OCS. This is auspicious because the neurotrophic factors regulate synaptic plasticity [52], and the reduction of resting epinephrine improves the autonomic nervous system function [53]. These elements could positively influence the cognitive, mood, and well-being aspects of older adults.

As to the psychoemotional level, there were no differences found between the OCS groups vs. control groups. However, the analysed studies reported improvements in diverse cognitive functions [37,41,43,45,46,48] and psychological aspects [42,43,48], while one study did not achieve any changes in the psychological variables [38]. What we found in our review is consistent with what is informed by a meta-analysis that revealed significant improvements concerning attention and cognitive processing speed in older adults intervened through resistance training and tai chi when compared to groups without PA [54]; however, the same authors suggest that the results must be interpreted with caution since the analysed studies presented several differences. Despite this, it has been proposed that improvements at a cognitive level could reduce or prevent cognitive impairment [2]; thereby, it is relevant to analyse different PA strategies, such as OCS, to design and prescribe PA programs that favor changes at the cognitive level [55].

The OCS interventions analysed in our review account for activities that lasted between eight and 24 weeks, with one to five weekly sessions of 60 to 90 min; intensity was only reported by four studies [37,38,39,44], which was moderate to vigorous. PA recommendations for older adults propose that the activities should maintain a moderate intensity and a minimum of three sessions per week [13]; in addition, it has been suggested that at least two sessions should include muscle-strengthening exercises focused on the major muscle groups [3]. Although our review’s analyses are not conclusive, we can state that the reported activities by OCS interventions do favor the general health of older adults, which agrees with current PA recommendations [13].

Another remarkable aspect concerning OCS interventions corresponds to the participants’ adherence rate. Ten studies reported adherence equal to or greater than 80% [37,38,39,41,42,43,44,46,47,48], whereas only two studies reported adherence close to 50% [40,45]. Overall, the main reasons for drop-out were related to diseases, personal problems, not meeting the training sessions’ minimum attendance, and conflicts with the training schedule, while only two studies included loss of interest [40,41] and foot injuries [41] into the reasons for drop-out. The adherence reflected by OCS interventions was greater than that informed by PA governmental programs for older adults [6], which reach 50%. In this sense, it is relevant to use PA strategies that are attractive to older adults to generate greater adherence. Moreover, it has been suggested that beyond establishing strict PA criteria, the most important goal is to avoid sedentary lifestyles and promote regular PA for older adults [56].

The main strengths of this review were: (i) the methods used for selecting and assessments the studies, which followed the PRISMA-P, PROSPERO, and TESTEX protocols recommendations, (ii) the inclusion of five generic databases (SCOPUS, PubMed/MEDLINE, Web of Science, PsycINFO, EBSCO) to gather information, increasing accuracy and decreasing possible biases of the results obtained, and (iii) the consideration of three languages (English, Spanish, and Portuguese) and a range higher of 30 years (1990 onwards) to select the studies, which widened the search scope. As limitations, we point out: (i) the diversity of instruments and variables observed, as well as the small number of high-methodological-quality studies available, which made a quantitative analysis (meta-analysis) of the data impossible, (ii) the lack of clarity regarding the distribution of the general activities included in the analysed studies (e.g., cardiorespiratory capacity, muscle strength, flexibility, etc.), as well as the scarce report of intensity (only four studies indicated it), which limits the replicability of the interventions, and (iii) not finding studies that used the fencing or wrestling modality as interventions, which reduces the generalization of the data. These facts supports that PA regarding the older population is an emerging field that needs more support and research [6]. Considering the results obtained in this systematic review, is it possible to prescribe or stimulate PA means by OCS in older adults. A choice among different PA modalities can be incorporated, focusing on improving general health status. Further, OCS could be an option to promote health if the participants had adherence, pleasure, and satisfaction. Thus, OCS’s practice will be one more means for self-care in health.

## 5. Conclusions

OCS interventions (i.e., boxing, judo, karate, and taekwondo) improve older adults’ physical-functional, physiological, and psychoemotional health. Our systematic review confirms that OCS training has high adherence (greater than 80%) in older adults.

Therefore, we recommend selecting OCS with non-contact activities, involving specific movements of the upper (punching and blocking) and lower extremities (stances, mobilization, and kicking), practiced individually or in pairs through choreographies or forms, adapted to the characteristics of older adults, following basic training principles (e.g., progressive overload), with a moderate to vigorous intensity and a frequency of two or three weekly sessions of 60 min. However, future studies could report in more detail the general and specific activities developed in OCS interventions for older adults, as well as the intensity of the exercises.

## Figures and Tables

**Table 1:** Study quality assessment according to TESTEX scale.

Study	Elegibility Criteria Specified	Randomly Allocated Participants	Allocation Concealed	Gorups Similar at Baseline	Assessors Blinded	Outcome Measures Assesed >85% of Participants *	Intention to Treat Analysis	Reporting of between Group Statistical Comparisons	Point Measures and Measures of Variability Reported **	Activity Monitoring in Control Group	Relative Exercise Intensity Reviewed	Exercise Volume and Energy Expended	Overall TESTEX #
Cho & Roh [37]	Yes	Yes	Yes	Yes	Unclear	Yes (2)	Yes	Yes	Yes (1)	No	Yes	Yes	11/15
Ciaccioni et al. [38]	Yes	Yes	Yes	Yes	Unclear	Yes (3)	Yes	Yes	Yes (2)	No	Yes	Yes	13/15
Ciaccioni et al. [39]	Yes	Yes	Yes	Yes	Unclear	Yes (3)	Yes	Yes	Yes (2)	Yes	Yes	Yes	14/15
Combs et al. [40]	Yes	Yes	Yes	Yes	No	Yes (3)	Yes	Yes	Yes (2)	Yes	No	Yes	13/15
Hu et al. [41]	Yes	Yes	Yes	Yes	No	Yes (3)	Yes	Yes	Yes (1)	Yes	No	Yes	12/15
Jansen & Dahmen-Zimmer [42]	Yes	Yes	Yes	No	Unclear	Yes (2)	Yes	Yes	Yes (1)	Yes	No	Yes	10/15
Jansen et al. [43]	Yes	Yes	Yes	Yes	Unclear	Yes (3)	Yes	Yes	Yes (2)	Yes	No	Yes	13/15
Lee et al. [44]	Yes	Yes	Yes	Yes	Unclear	Yes (2)	Yes	Yes	Yes (1)	Yes	Yes	Yes	12/15
Pacheco Lopes et al. [45]	Yes	Yes	Yes	Yes	Unclear	Yes (2)	Yes	Yes	Yes (1)	No	No	Yes	10/15
Pliske et al. [46]	Yes	Yes	Yes	Yes	Unclear	Yes (1)	Yes	Yes	Yes (1)	No	No	Yes	9/15
Witte et al. [47]	Yes	Yes	Yes	Yes	No	Yes (3)	Yes	Yes	Yes (2)	No	No	Yes	12/15
Witte et al. [48]	Yes	Yes	Yes	Yes	Unclear	Yes (3)	Yes	Yes	Yes (2)	No	No	Yes	12/15

* Three points possible: one point if adherence > 85%, one point if adverse events reported, one point if exercise attendance is reported. ** Two points possible: one point if primary outcome is reported, one point if all other outcomes reported. # total out of 15 points. TESTEX: Tool for the assEssment of Study qualiTy and reporting in Exercise [26].

**Table 2:** Characteristics of the studies that analyse the effects of Olympic combat sports on older adult’s health status.

Study	Country	Modality of OCS	Sample	Activities Developed in the Intervention	Training Volume			Training Intensity
					Total Duration (Weeks)	Frequency (Weekly)	Time Per Session (Min)	
Cho & Roh [37]	South Korea	Taekwondo	37 older women (mean age 68.9 years)					
			Taekwondo group (*n* = 19)	Stretching, basic taekwondo movement (stance, block, punch, strike, thrust). Poomsae (Taegeuk 1–4 chapter). Kicking (front kick, side kick, roundhouse kick, downward kick). Step (forward, side, backward). Practice mitt kicking. Taekwon gymnastics (2 music-based gymnastics).	16	5	60	Exercise at 50–80% HRmax
			Control group (*n* = 18)	They were asked to maintain their usual activities.	16	NR	NR	NR
Ciaccioni et al. [38]	Italy	Judo	40 older adults (age between 64 and 77 years)					
			Judo group (*n* = 19, 10 men and 9 women)	10-min judo-specific warm-up (e.g., light activities and gentle routines of judo postures, movements, and techniques performed at slow speed). 30-min judo (e.g., ne-waza—ground technique, tachi-waza—standing techniques, kata—sequences of specific movements, ukemi—judo falling techniques). A 20-min judo cooldown (e.g., judo kata focused on stretching and relaxation).	16	2	60	Moderate to vigorous
			Control group (*n* = 21, 12 men and 9 women)	They were asked to maintain their usual activities.	16	NR	NR	NR
Ciaccioni et al. [39]	Italy	Judo	30 older adults (mean age 69.7 years)					
			Judo group (*n* = 16, 8 men and 8 women)	10-min judo-specific warm-up (e.g., light routines and dynamic movements of the whole body mimicking the judo techniques). 30-min judo central part (e.g., standing, ground and ukemi breaking-fall techniques, as uchi-komi repetition training in couple and kata sequences of specific movements, individually and in couple). A 20-min cool-down (e.g., stretching and relaxation using judo techniques performed as kata).	15	2	60	Moderate to vigorous
			Control group (*n* = 14, 9 men and 5 women)	They were asked to maintain their usual activities.	15	NR	NR	NR
Combs et al. [40]	United States of America	Boxing	31 older adults (mean age 67.3 years)					
			Boxing group (*n* = 17, 11 men and 6 women)	Boxing-specific activities via a circuit training, as well as general endurance activities. Activities were self-progressed by encouraging participants to train as intensely as they could tolerate and by striving to complete more repetitions during each training bout. Participants wore boxing gloves and hit boxing-specific punching bags, but did not contact other people while boxing.	12	2–3	90	NR
			Traditional exercise group (*n* = 14, 10 men and 4 women)	Each traditional group exercise session began with a 15-min warm-up period consisting of various seated exercises such as multi-planar axial and extremity active range of motion and stretching. The next hour consisted of strengthening exercises, endurance training, and balance activities. Endurance activities included walking in- or out-doors at a self-selected pace and stair climbing. Static and dynamic standing balance activities were performed on different support surfaces and visual input (eyes open and closed). Ended with a 15 m seated cool-down similar to the warm-up plus breathing exercises for relaxation	12	2–3	90	NR
Hu et al. [41]	China	Boxing	198 older adults (mean age 70 years, 145 men and 53 women)					
			Boxing group (*n* = 96)	The program consisted of jogging for 30 min and shadowboxing for 60 min once a week.	24	1	90	NR
			Control group (*n* = 102)	NR	24	NR	NR	NR
Jansen & Dahmen-Zimmer [42]	Germany	Karate	45 older adults (mean age 78.8 years).					
			Karate group (*n* = 12, 4 men and 8 women).	Training was performed accordingly the guidelines of the German-Karate Federation. Long sequences of arm and leg movements were taught.	24	1	60	NR
			Physical exercise group (*n* = 12, 5 men and 7 women).	Training included simple exercises for strength, mobilization, stretching, and relaxation. Everyday objects such as towels, chairs, etc., were used as exercise equipment. Each session was preceded by warming-up and ended with cooling-down.	24	1	60	NR
			Cognitive group (*n* = 12, 5 men and 7 women	The program consists of 121 tasks, 104 inductive thinking tasks (13 generalizations, 15 discrimination, 17 cross-classification, 32 relations, 14 relation differentiation, and 13 system-building tasks), and 17 deductive thinking and concentration tasks. Tasks appear in a specific order to allow the participants to become familiarized with the task.	24	1	60	NR
			Control group (*n* = 9, 1 men and 8 women).	NR	24	NR	NR	NR
Jansen et al. [43]	Germany	Karate	54 older adults (mean age 63.5 years).					
			Karate group (*n* = 23, 6 men and 17 women)	Karate-Do involves Kihon, Kumite, and Kata. Kihon involves performing specific movements with legs or arms and combinations of both, kumite consists of training with a partner, kata is the execution of a variety of tactical fighting exercises. Participants learned simultaneous movements of legs and arms and exercised partner training.	8	2	60	NR
			Mindfulness-based stress reduction (*n* = 14, 6 men and 8 women)	The MBSR is composed of didactic and practice elements. It incorporates sitting and walking meditation, body scan exercises, and mindfulness communication. Training focuses on the perception of and attention to one’s own body, mental states, and emotions in all these elements.	8	2	60	NR
			Control group (*n* = 17, 9 men and 8 women)	NR	8	NR	NR	NR
Lee et al. [44]	South Korea	Taekwondo	20 older women (mean age 70 years)					
			Taekwondo group (*n* = 10)	Dynamic stretching warm-up for 10 min, then Taekwondo training for 40-min. This consisted of kicks, punches, steps and step-sparring while facing an opponent. They then spent the remaining time practicing Taekwondo forms and then walked, jogged or ran, depending on what intensity was desired. A 10-min, static stretching cool-down.	12	3	60	30–40% HRmax (4-w). 40–50% HRmax (4-w). 50–60% HRmax (4-w).
			Control group (*n* = 10)	They were asked to maintain their usual activities.	12	3	60	NR
Pacheco Lopes et al. [45]	Brazil	Karate	33 older adults (mean age 68.7 years)					
			Karate group (*n* = 16, 1 men and 15 women)	Training session consisted of brief warmup (5–10 min); Kihon exercises, kata (sequences of Karate-Do movements), kumite and breathing techniques (40–45 min) and relaxation through brief meditation exercises tailored to the needs of the participants (10 min).	12	2	60	NR
			Control group (*n* = 17, 1 men and 16 women).	They were asked to maintain their usual activities.	12	NR	NR	NR
Pliske et al. [46]	Germany	Karate	68 older adults (mean age 69 years, 29 men and 39 women).					
			Karate group (*n* = 25)	Training session consisted of basic techniques and katas. Kumite and self-defence were no components of the training. The training were adapted to the subjects’ age.	20	2	60	NR
			Fitness group (*n* = 24)	Specific exercises for balance, strength and coordination as well as simple team sports and games were practiced. The individual exercises, except for sports games, had a non-competitive character.	20	2	60	NR
			Control group (*n* = 19)	They were asked to maintain their usual activities.	20	NR	NR	NR
Witte et al. [47]	Germany	Karate	90 older adults (mean age 69.3 years, 35 men and 55 women).					
			Karate group (*n* = 30)	The training was as follows: several stances (forward stance, back stance, and straddle-leg stance), several arm techniques during standing, and forward and backward stances (downward block, lunge punch, reverse punch), and upper blocks. Furthermore, the participants learned simple attacks and defense exercises with their partners and a simple kata.	20	2	60	NR
			Fitness group (*n* = 30)	The fitness training included elements of gymnastics, running exercises, practices with a ball and other hand devices, and strengthening exercises.	20	2	60	NR
			Control group (*n* = 30)	They were asked to maintain their usual activities.	20	NR	NR	NR
Witte et al. [48]	Germany	Karate	89 older adults (mean age 70 years, 36 men and 53 women)					
			Karate group (*n* = 30)	They included stances such as forward stances, back stances, and straddle-leg stances to train leg and trunk musculature and balance skills; also included were several arm techniques in standing positions and forward and backward walking (downward-block), lunge punches, reverse punches, and upper blocks to improve arm–leg coordination, and a special karate form	20	2	60	NR
			Fitness group (*n* = 30)	The training unit contained elements of gymnastics, running exercises, practices with balls and other hand devices, age-related games and strengthening exercises.	20	2	60	NR
			Control group (*n* = 29)	They were asked to maintain their usual activities.	20	NR	NR	NR

HRmax: maximum heart rate. NR: not reported. MBSR: mindfulness-based stress reduction; Modality of OCS: practiced Olympic combat sports. Sample: total number, mean age of participants, intervention groups and gender. W = weeks.

**Table 3:** Effects reported by studies with Olympic combat sports on older adults’ health status.

Study	Analysed Variables	Data Collection Instruments	Main Outcomes
Cho & Roh [37]	**Physical-functional level** Muscle strength (lower body). Muscle strength (upper body). Flexibility (lower body). Flexibility (upper body). Endurance fitness. Agility and dynamic balance.	Chair stand test Arm curl test Chair sit-and-reach test Back scratch test 2-min step test Timed Up-and-go test	There significant increases (*p* < 0.05) in chair stand test, chair sit-&-reach test, 2-min step test, in the level of BDNF, VEGF, and IGF-1, and the Color-Word component score in the taekwondo group, without reporting changes in the rest of the variables. Furthermore, there were no significant changes in any of the variables in the control group, nor were there differences between the groups.
	**Physiological level** Serum levels of neurotrophic growth factors (BDNF, VEGF, IGF-1; systolic, diastolic, and mean blood flow velocity and pulsatility index of the middle cerebral artery).	ELISA kit	
	**Psychoemotional level** Cognitive functions	MMSE-DS. Stroop colour and word test	
Ciaccioni et al. [38]	**Physical-functional level** *Anthropometric measurements* Body weight. Height. Body mass index. Wrist circumference. Hip circumference.	Digital scale Stadiometer kg/m^2^ Measuring tape (cm) Measuring tape (cm)	The judo group showed reductions of waist circumference (Δ = −1%, d = 0.2) and improvements for lower and upper body flexibility (Δ = +69%, d = 0.4 and Δ = +126%, d = 0.5, respectively) and strength (Δ = +12%, d = 0.6 and Δ = +31%, d = 1.6, respectively). The control group showed a decline in lower body strength (Δ = −12%, d = 0.8). Psychological variables did not reveal significant effects.
	*Physical-functional measurements* Grip strength. Muscle strength (lower body). Muscle strength (upper body). Flexibility (lower body). Flexibility (upper body). Inter-limb coordination. Low risk of falls.	Handgrip dynamometer Chair stand test Arm curl test Chair sit-and-reach test Back scratch test Digital metronome Berg balance scale	
	**Psychoemotional level** Body image perception. Quality of life perception. Fear of falling.	Body image dimensional assessment SF-12v2 FES-I	
Ciaccioni et al. [39]	**Physical-functional level** Gait stability (flat and hurdling conditions: gait cycle, speed, and cadence).	10-m Optojump photocell system	A significant Time × Motor complexity × Group interaction was found for mean values and coefficients of variation. For mean values, Judo group showed improvements for flat and hurdling conditions in step length (flat: ∆ = +2.6%, d = 0.4; hurdling: ∆ = +3.2%, d = 0.4), gait cycle (flat: ∆ = −4.3%, d = 0.4; hurdling: ∆ = −4.0%, d = 0.5), speed (flat: ∆ = +6.6%, d = 0.7; hurdling: ∆ = +6.7%, d = 0.6) and cadence (flat: ∆ = 4.3%, d = 0.4; hurdling: ∆ = 3.9%, d = 0.5). For coefficients of variation, Judo group improved step length for flat (∆ = −20.9%, d = 0.6) and hurdling (∆ = −16.3%, d = 0.8) conditions, whereas control group showed a deterioration in the step length for the corridor condition (∆ = +22.3%, d = 0.7).
Combs et al. [40]	**Physical-functional level** Low risk of falls. Agility and dynamic balance. Agility and dynamic balance. Gait velocity. Endurance fitness. Activities-specific balance.	Berg balance scale Timed up-and-go test Dual-task timed up-and-go test Gait velocity 6-min Walk test ABC	The traditional exercise group demonstrated significantly greater gains in balance confidence than the boxing group (*p* < 0.025). Only the boxing group demonstrated significant improvements in gait velocity and endurance over time with a medium between-group effect size for the gait endurance (d = 0.65). Both groups demonstrated significant improvements with balance, mobility, and quality of life with large within-group effect sizes (d ≥ 0.80).
	**Psychoemotional level** Quality of life perception.	PDQL	
Hu et al. [41]	**Physical-functional level** Activity of Daily Living. Body movement testing.	AVD scale DDX-200 computer multifunction tester	Compared with control group, patients who exercised showed improved cognitive function in immediate memory (*p* < 0.001) and delayed recall (*p* = 0.004) function. In addition, activities associated with daily living showed improvement (*p* < 0.001), as did body movement (*p* < 0.05), arm stability (*p* < 0.001), and the appearance of rotation (*p* < 0.05).
	**Psychoemotional level** Cognitive function.	Chinese MMSE	
Jansen & Dahmen-Zimmer [42]	**Psychoemotional level** Cognitive speed. Memory performance.	ZVT and ZS Digit-span test. Figure test. Block-tapping test	No significant difference in cognitive improvement dependent on group between the three training conditions. However, a significant improvement was found in the emotional mental state measurement for the Karate group compared to the waiting control group.
	Emotional mental state and depressive pathology.	CES-D	
Jansen et al. [43]	**Physiological level** Perceived stress. Chronic stress.	TICS Hair cortisol concentration	Significant improvement (*p* < 0.05) for the karate group, but not the mindfulness-based stress reduction and control group, in subjective mental health and anxiety as well as cognitive processing speed. The mindfulness-based stress reduction group showed by trend as a decrease in stress. No significant correlation between pre assessment hair cortisol and post-assessment outcomes could be established. However, the higher the level of baseline self-reported perceived stress, the higher the increase in depression, anxiety, and chronic stress.
	**Psychoemotional level** *Psychological measurements* Emotional well-being. Anxiety and depression. Optimism and pessimism. Quality of life perception.	MDBF HADS LOT-R SF-12	
	*Cognitive measurements* Mental rotation. Cognitive speed. Inhibition and Memory performance	The mental rotation test ZVT Stroop colour and word test. Digit-span test	
Lee et al. [44]	**Physical-functional level** Muscle strength (lower body). Grip strength.	1RM a leg extension machine Handgrip dynamometer	There were significant (*p* < 0.05) group by time interactions for resting epinephrine and Norepinephrine levels, with resting epinephrine decreasing in the taekwondo training group and norepinephrine increasing in the control group. Additionally, brachial-ankle pulse wave velocity, resting heart rate, and blood pressure were significantly decreased, while handgrip and leg strength were significantly increased in the taekwondo training group compared to the control group.
	**Physiological level** Blood catecholamine levels of epinephrine and norepinephrine.	ELISA kit	
	Brachial-ankle pulse wave velocity.	Non-invasive arterial tonometry with SphygmoCor	
Pacheco Lopes et al. [45]	**Physical-functional level** Motor sequencing capacity through hand movements.	Motor Task Sequence	Karate group shows significantly (*p* < 0.05) better results than the control group in visual memory tasks, executive functions, and memory complaints in post-intervention analysis.
	**Psychoemotional level** Cognitive functions. Divided attention and visual processing speed. Concentrated attention and memory performance. Visual perception, and memory. Episodic verbal memory and recognition. Object-centered attention and visuospatial memory.	MMSE Trail making test Digit Span test Rey-Osterrieth complex figure Rey Auditory-Verbal Learning Test Visual Memory Span test Wisconsin Card Sorting Test. Verbal Fluency	
	Executive functioning, cognitive flexibility, lexical production and semantic memory. Depressive symptoms. The intensity of anxiety. Subjective complaints of memory.	Geriatric depression scale Beck Anxiety Inventory APT-II Attention Questionnaire PRMQ	
	Difficulties in prospective and retrospective memory. Complaints of memory problems. Subjective assessment of executive functions	Memory Complaint Questionnaire Dysexecutive Questionnaire	
Pliske et al. [46]	**Physical-functional level** Normal walk. Motor dual task.	kinematic system for motion analysis	It could be seen that all groups significantly improved (*p* < 0.05) their gait parameters after a 5-month period, even the control group. A sporty intervention seems to affect mainly the temporal gait parameters positively. This effect was especially demonstrated for a normal walk and dual cognitive task.
	**Psychoemotional level** Cognitive dual task.	kinematic system for motion analysis	
Witte et al. [47]	**Physical-functional level** Muscle strength (lower body). Walk. Static and dynamic balance.	Chair stand test Walking speed Balance ability	Significant improvements (*p* < 0.01) in performance in the chair stand test were found in the three groups, but in the karate group it has a greater effect. The karate group and control group show significant improvements in walking speed (*p* < 0.05), but the improvement in the karate group is greater (*p* < 0.01). Significant changes (*p* < 0.05) in balance ability were found in the karate group and the control group.
Witte et al. [48]	**Physical-functional level** Motor reaction.	The rod test	Significant improvement (*p* < 0.10) in motor reactivity, stress tolerance, and divided attention only after the 5-month karate training period. Additionally, the results of the secondary study indicate further improvements after 10 months.
	**Psychoemotional level** Cognitive performance. Reactive stress tolerance. Divided attention.	The DemTect test Determination Test Version S11 Test of Divided Attention	

ABC: Activities-specific balance confidence scale. ADL: The Activity of daily living. BDNF: Brain-derived neurotrophic factor. CES-D: The Center of Epidemiological Studies Depressions Scale, long version. ELISA: enzyme-linked immunosorbent assay. FES-I: The Falls Efficacy Scale-International. HADS: The Hospital Anxiety and Depression Scale. IGF-1: insulin-like growth factor-1. LOT-R: The Life Orientation Test–Revised. MDBF: The Multidimensional Mood Questionnaire. MMSE: The Mini-Mental State Examination. MMSE-DS: The Mini-Mental State Examination for dementia screening. PDQL: Parkinson’s disease quality of life scale. PRMQ: Prospective and Retrospective Memory Questionnaire. SF12: The Short form Health Survey. SF12v2: The Short form Health Survey, version 2. TICS: the Trier Inventory for Chronic Stress. VEGF: vascular endothelial growth factor. ZS: Number–Symbol Test. ZVT: Number-connection Test. 1RM: one-repetition maximum test.

## Data Availability

The datasets generated during and/or analysed during the current review are available from the Corresponding author on reasonable request.
